# Cancer burden in Europe: a systematic analysis of the GLOBOCAN database (2022)

**DOI:** 10.1186/s12885-025-13862-1

**Published:** 2025-03-12

**Authors:** Mohammed Elmadani, Peter Onchuru Mokaya, Ahmed A. A. Omer, Evans Kasmai Kiptulon, Simon Klara, Mate Orsolya

**Affiliations:** 1 Doctoral School of Health Sciences, Faculty of Health Sciences, University of Pecs, Vorosmarty Mihaly Street 4, Pecs, 7621 Hungary; 2 Jamhuriya Research Center, Jamhuriya University of Science and Technology, Mogadishu, Somalia; 3 Department of Epidemiology, Faculty of Public Health, University of El Imam El Mahdi, Kosti, Sudan; 4 Institute of Clinical Pharmacy, University of Szeged, Szeged, Hungary

**Keywords:** Age-standardized rates (ASRs), Cancer incidence, Cumulative risk, Cancer mortality, And Cancer inequity

## Abstract

**Background:**

Cancer remains a significant public health challenge in Europe, with substantial regional disparities in incidence, mortality, and access to healthcare. This study analyses cancer patterns across Eastern, Northern, Southern, and Western Europe in 2022, highlighting key public health implications and gaps in prevention and treatment.

**Methods:**

Using data from GLOBOCAN 2022, this study assessed total new cancer cases, age-standardized incidence and mortality rates (ASRs) per 100,000, and cumulative cancer risk at age 75. The top three cancers by sex and region were also analysed to identify trends and disparities.

**Results:**

In 2022, Europe recorded 4,471,422 new cancer cases (ASR 280 per 100,000), with a cumulative risk of 27.9% by age 75. Males accounted for 2,359,303 cases (ASR 319.6, cumulative risk 31.9%), while females had 2,112,119 cases (ASR 253.4, cumulative risk 24.7%). Northern and Western Europe had the highest incidence rates, with Denmark leading at 374.7 per 100,000 (cumulative risk 34.9%), likely due to advanced screening and healthcare. Conversely, Eastern Europe had the highest mortality, with 1,091,871 deaths (ASR 135.3), reflecting late diagnoses and limited access of treatment. Hungary exhibited the highest mortality rate (ASR 143.7, cumulative risk 15.8%), followed by Poland (ASR 133.1). Prostate and breast cancers were the most common in males and females, respectively. Lung cancer, despite a lower incidence (ASR 24.7), had the highest mortality (ASR 17.7), while pancreatic cancer showed high fatality (ASR 6.3, mortality ASR 5.6). Thyroid cancer had a relatively high incidence (ASR 7.5) but low mortality (ASR 0.21).

**Conclusions:**

Significant regional disparities in cancer burden underscore the need for targeted public health strategies. Expanding cancer screening programs, strengthening smoking cessation and HPV vaccination efforts, and improving healthcare accessibility particularly in Eastern Europe are critical to reducing mortality and enhancing early detection. Differences in mortality-to-incidence ratios also highlight the role of healthcare infrastructure and timely interventions. Future research should explore the socioeconomic and environmental determinants driving these disparities to inform evidence-based cancer control policies across Europe.

## Background

Cancer is the second leading cause of death worldwide and presents a significant public health challenge, particularly in Europe, which, despite representing only 9% of the global population, accounts for 20.3% of cancer-related deaths and 23.4% of diagnoses [1–5]. According to the European Cancer Information System, approximately 2.74 million new cancer cases were reported in 2022, with projections indicating that this number could exceed 3.24 million by 2040 [2]. The leading cancers include breast, prostate, lung, and Colorectum cancers, which together account for approximately half of all new cases [6]. This increase is largely attributed to an aging population, as older age groups are more susceptible to cancer [6–8]. In 2022, approximately 1.29 million cancer deaths were reported, with significant trends and disparities observed between Western Europe and Eastern Europe [2]. Key lifestyle factors, including tobacco use, poor diet, physical inactivity, and environmental exposures, are closely linked to elevated cancer rates [1, 9]. Lung cancer, which is particularly prevalent due to high smoking rates in Central and Eastern Europe, is responsible for more than 384,000 deaths annually [2, 10]. The Global Burden of Disease study revealed a 91.8% increase in lung cancer deaths from 1990 to 2019, primarily due to demographic changes rather than improvements in smoking cessation [10]. Breast cancer, the most frequently diagnosed cancer among women, represents approximately 30% of all cancer cases, with an incidence rate of 463.2 cases per 100,000 people in 2019, which is 1.7 times greater than the global average [11–13]. Regional disparities further define Europe’s cancer burden. Central and Eastern European countries, such as Hungary and Latvia, report higher cancer mortality rates than their Western and Northern counterparts do, reflecting differences in healthcare quality, early detection, and socioeconomic conditions [14–16]. These disparities are influenced by a complex interplay of factors, including healthcare access, socioeconomic status, screening programs, and public health policies which influence cancer outcomes across different populations [14, 17]. Countries with well-established screening and early detection programs often report higher incidence rates due to increased case identification, yet they experience lower mortality rates because of timely and effective treatments. For example, nations with organized cervical cancer screening and comprehensive HPV vaccination programs have seen significant reductions in both incidence and mortality rates [18]. Conversely, regions with limited access to healthcare services may report lower incidence rates, potentially reflecting underdiagnosis rather than a true lower disease burden. Economic disparities further exacerbate these differences, as resource-limited settings often face challenges such as delayed diagnoses and restricted access to advanced treatments, leading to higher mortality rates. Policy differences also play a crucial role; countries with robust tobacco control measures, for instance, tend to have lower rates of smoking-related cancers [19]. Addressing these disparities necessitates a comprehensive approach that includes equitable access to screening programs, preventive vaccinations, and targeted public health interventions.

Despite these challenges, Europe has made remarkable progress in cancer research and treatment over the past two decades [20]. Population-based screening programs, such as mammograms and colonoscopies, have facilitated earlier cancer detection, significantly improving survival rates [20]. Advanced technologies, including microfluidics and AI, increase the accuracy and efficiency of screening processes, allowing timely interventions [21]. Precision medicine and targeted therapies have revolutionized treatment, particularly for challenging cancers such as lung cancer and melanoma [22]. Additionally, immunotherapies have emerged as effective options, providing personalized care that aligns with individual patient profiles [22]. To address the growing cancer burden in Europe, key priorities include reducing risk factors (e.g., tobacco use and obesity) and expanding vaccination efforts, particularly for HPV, to lower cervical cancer rates. Ensuring equitable access to screening and advanced treatments across all regions is crucial for minimizing survival disparities [23].

While GLOBOCAN 2022 provides comprehensive global and regional estimates of cancer incidence and mortality [4], its analyses often present aggregated data without an in-depth exploration of the disparities within and between European regions. This study goes beyond these general estimates by conducting a comparative analysis of cancer incidence and mortality across different European regions, highlighting the impact of healthcare accessibility, screening programs, and socioeconomic disparities on cancer outcomes. By identifying variations in cancer burden across countries and correlating them with contextual factors such as smoking prevalence, vaccination coverage, and healthcare infrastructure, this study aims to fill a critical gap in the literature. Understanding these regional differences is essential for tailoring public health interventions and informing policies that address disparities in cancer prevention and care.

## Methods

This study presents a systematic analysis of the cancer burden across Europe, focusing on incidence and mortality. The data for this analysis were obtained from GLOBOCAN 2022, compiled by the World Health Organization’s International Agency for Research on Cancer (IARC) and presented in the Global Cancer Observatory (GCO) database (https://gco.iarc.who.int). This resource provides comprehensive information on cancer cases from 40 European countries, with a focus on the most recent available year, 2022. The sources of cancer incidence data varied across Europe. In Northern Europe, the data primarily came from national cancer registries (Category 1). In contrast, incidence data were estimated using national mortality data through modelling in Eastern and Southern Europe. These estimates were based either on mortality-to-incidence ratios derived from country-specific cancer registries (Category 3a) or, when such data were unavailable, from cancer registries in neighbouring countries (Category 3b) [24]. The reporting discrepancies and lack of data in cancer statistics across Europe present significant challenges for public health and cancer control initiatives. These issues arise from data quality deficiencies, harmonization challenges among registries, and socio-economic disparities affecting data collection and reporting. Common data quality issues include missing data, class imbalances, and timeliness problems, which limit the applicability of real-world data in oncology [25]. Population-based cancer registries (PBCRs) also struggle with data comparability due to inconsistent data collection and processing methods across different regions [26]. Despite ongoing efforts to improve data quality and harmonization among PBCRs, discrepancies persist due to national data protection regulations and differences in healthcare systems [27]. The European Cancer Inequalities Registry (ECIR) aims to monitor and address these disparities, highlighting significant socio-economic and demographic inequalities in cancer care [28]. Socio-economic disparities further contribute to variations in cancer outcomes, as access to screening, healthcare quality, and health education differ across European countries [29]. For instance, participation in breast cancer screening remains notably lower in certain demographics, impacting survival rates [28].

The GCO website facilitates the tabulation and graphic visualization of the GLOBOCAN database at the global, regional, and national levels, categorized by cancer type, sex, and age. The study encompasses the entire population of the European continent, including all age groups and genders, covering approximately 750 million individuals. The analysis included all EU countries in GLOBOCAN 2022. Estimates are available in the GCO for 33 cancer types based on the International Classification of Diseases, Tenth Edition (ICD-10). The numbers of new cancer cases and cancer deaths were extracted for all cancers combined (ICD-10 codes C00–97/C44) and for 33 cancer types: Lip, oral cavity (C00-06), Salivary glands (C07-08), Oropharynx (C09-10), Nasopharynx (C11), Hypopharynx (C12-13), Esophagus (C15), Stomach (C16), Colorectum (C18-C21), Liver (Including intrahepatic bile ducts) (C22), Gallbladder (C23), Pancreas (C25), Larynx (C32), Lung (including Trachea and bronchus) (C33-34), Melanoma (C43), Mesothelioma (C45), Kaposi sarcoma (C46), Breast (C50), Vulva (C51), Vagina (C52), Cervix uteri (C53), Corpus uteri (C54), Ovary (C56), Penis (C60), Prostate (C61), Testis (C62), Kidney (C64), Bladder (C67), Brain, central nervous system (C70-72), Thyroid (C73), Hodgkin lymphoma (C81), non-Hodgkin lymphoma (C82-86 + C88), Multiple myeloma (C90), and Leukaemia (C91-95).

This study identified the three most prevalent cancers and their associated mortality rates across Europe. In addition to the number of new cases and deaths, two measures of direct standardization are employed to facilitate comparisons between populations, adjusted for differences in age structures: age-standardized rates (ASRs) per 100,000 person-years and the cumulative risk of developing or dying from cancer before the age of 75 years. Cumulative risk represents the probability of an individual developing or dying from a disease within a specified period. In the context of cancer, it is typically expressed as the estimated number of newborns (per 100) who develop or die from a specific cancer over their lifetime (commonly up to age 74). This estimate assumes that individuals experience the cancer rates observed during the study period and that no other causes of death interfere [30]. Operationally, cumulative risk is calculated by dividing the number of new disease cases during the specified time interval by the population at risk at the beginning of the interval. This calculation provides a proportion that reflects the risk of developing the disease over that time frame [31].

Age significantly impacts cancer outcomes across different regions and countries, influenced by socioeconomic factors, healthcare access, and demographic trends. As populations age, the burden of cancer, particularly among older adults, is expected to rise, with disparities evident in incidence and mortality rates based on geographic and socioeconomic contexts [32, 33].

Analyses of cumulative cancer risk often focus on the period up to age 75 due to several interrelated factors. Firstly, cancer incidence rates appear to decline after ages 75 to 85 for most solid tumors, a trend largely attributed to reduced diagnostic efforts in the elderly, possibly due to underreporting or underdiagnosis, rather than a true decrease in cancer occurrence. Consequently, data beyond age 75 may not accurately reflect actual cancer risk, leading researchers to limit analyses to this age threshold [34]. Secondly, as individuals age, the likelihood of death from causes other than cancer rises significantly. This increase in competing mortality diminishes the probability that an individual will develop or succumb to cancer, as they may pass away from other conditions first. Therefore, focusing on cumulative risk up to age 75 provides a clearer picture of cancer risk without the confounding effect of other mortality factors [35]. Additionally, many cancer screening programs and preventive strategies are recommended up to age 75. Beyond this age, the benefits of routine screenings become less clear due to the increased risks associated with diagnostic procedures and the reduced life expectancy. As a result, cumulative risk assessments often align with these guidelines, concentrating on the age range where preventive interventions are most beneficial [36].

The data were stratified by country, cancer type, sex, and geographical region (western, northern, central, and eastern Europe). This study utilized publicly available data from GLOBOCAN 2022; therefore, ethical approval was not needed. However, the study adhered to the Declaration of Helsinki and ethical guidelines for secondary data analysis.

## Results

### Cancer incidence

The total European population (747.5 million) reported 4,471,422 new cases, an ASR of 280, and a cumulative risk of 27.9%. Breast, Colorectum, and lung cancers were the most common cancers across both sexes Table 1. The male population (361.2 million) recorded 2,359,303 new cancer cases, yielding an age-standardized rate (ASR) of 319.6 per 100,000 and a cumulative risk of 31.9%. The most common cancers among males were prostate, lung, and Colorectum cancer. Among females (386.3 million), 2,112,119 new cancer cases were observed, with an ASR of 253.4 and a cumulative risk of 24.7%. The leading cancers in females were breast, Colorectum, and lung cancers.

**Table 1:** Cancer incidence and mortality in Europe (2022): regional, Sex-Based, and leading Cancer trends

Region	Gender	Population	Incidence					Mortality			
			New cases*	Age-standardized rate	Cum. Risk (75 years)	Top 3 leading cancers (Ranked by Cases) **		Cancer deaths*	Age-standardized mortality rate	Cum. Risk (75 years)	Top 3 leading cancers (Ranked by Cases) **
**Eastern Europe**	Male	137,314,603	680,411	295.9	31	Prostate, Lung, Colorectum		380,569	159.6	17.8	Lung, Colorectum, Prostate
	Female	154,548,370	680,908	226.3	22.8	Breast, Colorectum, Corpus uteri		316,826	87.5	9.8	Breast, Colorectum, Lung
	Both Sexes	291,862,973	1,361,319	250.4	26.1	Breast, Colorectum, Lung		697,395	115.9	13.2	Lung, Colorectum, Breast
**Northern Europe**	Male	52,963,352	386,085	337.9	32.6	Prostate, Lung, Colorectum		149,838	111.7	11	Lung, Prostate, Colorectum
	Female	54,164,287	335,992	293	27.9	Breast, Colorectum, Lung		131,791	85.8	8.9	Lung, Colorectum, Breast
	Both Sexes	107,127,639	722,077	312.5	30.1	Prostate, Breast, Lung		281,629	97.4	9.9	Lung, Colorectum, Prostate
**Southern Europe**	Male	74,143,121	531,344	311	31.1	Prostate, Lung, Colorectum		250,044	124	12.8	Lung, Prostate, Colorectum
	Female	77,501,382	440,855	247.6	23.9	Breast, Colorectum, Lung		191,353	77	8	Lung, Breast, Colorectum
	Both Sexes	151,644,503	972,199	275.1	27.3	Breast, Colorectum, Lung		441,397	98.1	10.3	Lung, Breast, Colorectum
**Western Europe**	Male	96,820,948	761,463	338.2	33.1	Prostate, Lung, Colorectum		311,420	121.7	12.6	Lung, Prostate, Colorectum
	Female	100,087,764	654,364	277.1	26.6	Breast, Colorectum, Lung		254,252	82.8	8.8	Lung, Breast, Colorectum
	Both Sexes	196,908,712	1,415,827	304	29.7	Breast, Colorectum, Lung		565,672	100.4	10.6	Lung, Breast, Colorectum
**All Europe**	Male	361,242,024	2,359,303	319.6	31.9	Prostate, Lung, Colorectum		1,091,871	135.3	14.3	Lung, Prostate, Colorectum
	Female	386,301,803	2,112,119	253.4	24.7	Breast, Colorectum, Lung		894,222	84.4	9	Lung, Breast, Colorectum
	Both Sexes	747,543,827	4,471,422	280	27.9	Breast, Colorectum, Lung		1,986,093	106.3	11.5	Lung, Breast, Colorectum

* Includes nonmelanoma skin cancer (NMSC)

** NMSC is included in other cancers

### Eastern Europe

In Eastern Europe, the total population of 291.9 million had 1,361,319 new cases, with an ASR of 250.4 and a cumulative risk of 26.1%. The males (137.3 million) experienced 680,411 new cancer cases, with an ASR of 295.9 and a cumulative risk of 31%. The predominant cancers were prostate, lung, and Colorectum cancer. A total of 680,908 new cases were recorded for females (154.5 million), yielding an ASR of 226.3% and a cumulative risk of 22.8%. Breast, Colorectum, and corpus uteri cancers were most common in females.

### Northern Europe

In Northern Europe, the population of 107.1 million accounted for 722,077 cases, with an ASR of 312.5 and a cumulative risk of 30.1%, and the males (52.9 million) had 386,085 new cases, resulting in an ASR of 337.9 and a cumulative risk of 32.6%. Prostate, lung, and Colorectum cancers were the most common. Among females (54.2 million), 335,992 cases were reported, with an ASR of 293 and a cumulative risk of 27.9%.

### Southern Europe

In southern Europe, the overall population of 151.6 million experienced 972,199 cases, with an ASR of 275.1 and a cumulative risk of 27.3%, with males (74.1 million) reporting 531,344 new cases, with an ASR of 311 and a cumulative risk of 31.1%. The leading cancers were prostate, lung, and Colorectum cancer. A total of 440,855 cases were reported in females (77.5 million), with an ASR of 247.6% and a cumulative risk of 23.9%.

### Western Europe

The total population of 196.9 million in Western Europe had 1,415,827 new cases, with an ASR of 304 and a cumulative risk of 29.7%; among them, males (96.8 million) reported 761,463 new cancer cases, with an ASR of 338.2 and a cumulative risk of 33.1%. The most common cancers are prostate, lung, and Colorectum cancer. Among females (100.1 million), 654,364 new cases were observed, yielding an ASR of 277.1 and a cumulative risk of 26.6%.

### Cancer mortality

Across all of Europe, males experienced 1,091,871 cancer deaths, with an ASR of 135.3 and a cumulative risk of 14.3%. The leading causes of death were lung, prostate, and Colorectum cancers. Among females, 894,222 deaths were observed, yielding an ASR of 84.4 and a cumulative risk of 9%. In total, the number of cancer deaths across both sexes was 1,986,093, with an ASR of 106.3 and a cumulative risk of 11.5%. The most frequent causes of death were lung, breast, and Colorectum cancers Table 1.

### Eastern Europe

In Eastern Europe, there were 380,569 male cancer deaths, with an ASR of 159.6% and a cumulative risk of 17.8%. The leading causes of death were lung, Colorectum, and prostate cancers. Female cancer deaths numbered 316,826, with an ASR of 87.5 and a cumulative risk of 9.8%. Breast, Colorectum, and lung cancers were the most common causes of death. Overall, the region reported 697,395 deaths, with an ASR of 115.9 and a cumulative risk of 13.2%.

### Northern Europe

In Northern Europe, males experienced 149,838 cancer deaths, with an ASR of 111.7 and a cumulative risk of 11%. Lung, prostate, and Colorectum cancers are the leading causes of death. Among females, 131,791 deaths were recorded, with an ASR of 85.8 and a cumulative risk of 8.9%. Lung, Colorectum, and breast cancers were the most common. In total, there were 281,629 cancer deaths in Northern Europe, with an ASR of 97.4 and a cumulative risk of 9.9%.

### Southern Europe

In southern Europe, 250,044 cancer deaths were recorded in males, with an ASR of 124 and a cumulative risk of 12.8%. Lung, prostate, and Colorectum cancers are the leading causes of death. Females experienced 191,353 cancer deaths, with an ASR of 77 and a cumulative risk of 8%. Lung, breast, and Colorectum cancers were the most common. Overall, the region experienced 441,397 cancer deaths, with an ASR of 98.1 and a cumulative risk of 10.3%.

### Western Europe

In Western Europe, males had 311,420 cancer deaths, with an ASR of 121.7% and a cumulative risk of 12.6%. The leading causes of death were lung, prostate, and Colorectum cancers. Among females, 254,252 deaths were recorded, yielding an ASR of 82.8% and a cumulative risk of 8.8%. Lung, breast, and Colorectum cancers were the most common. In combination, the region experienced 565,672 deaths, with an ASR of 100.4 and a cumulative risk of 10.6%.

### Key trends in incidence and mortality rates

**Fig. 1:**
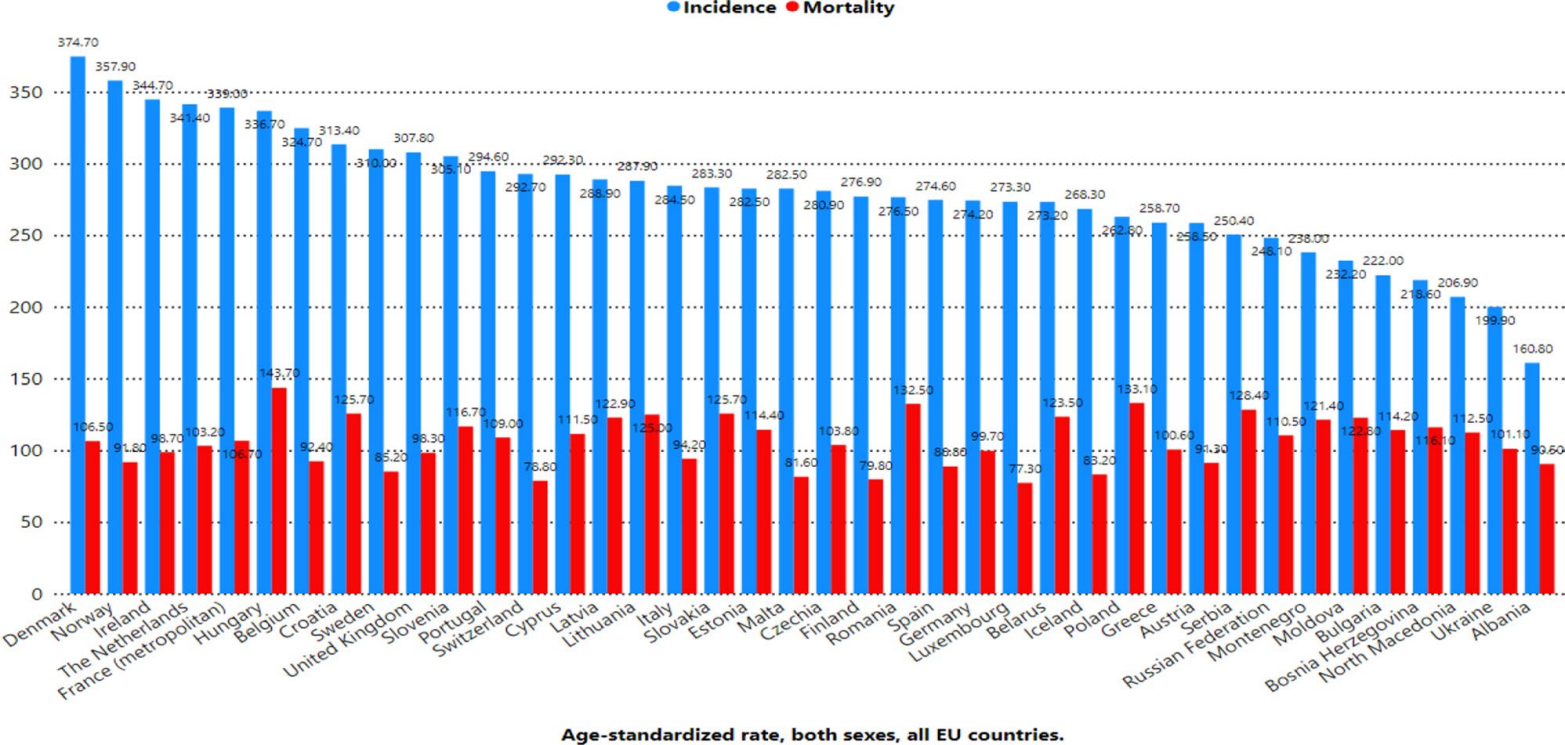
Age-standardized rate (world) per 100,000, incidence and mortality, both sexes, in 2022, all cancer, across Europe

**Fig. 2:**
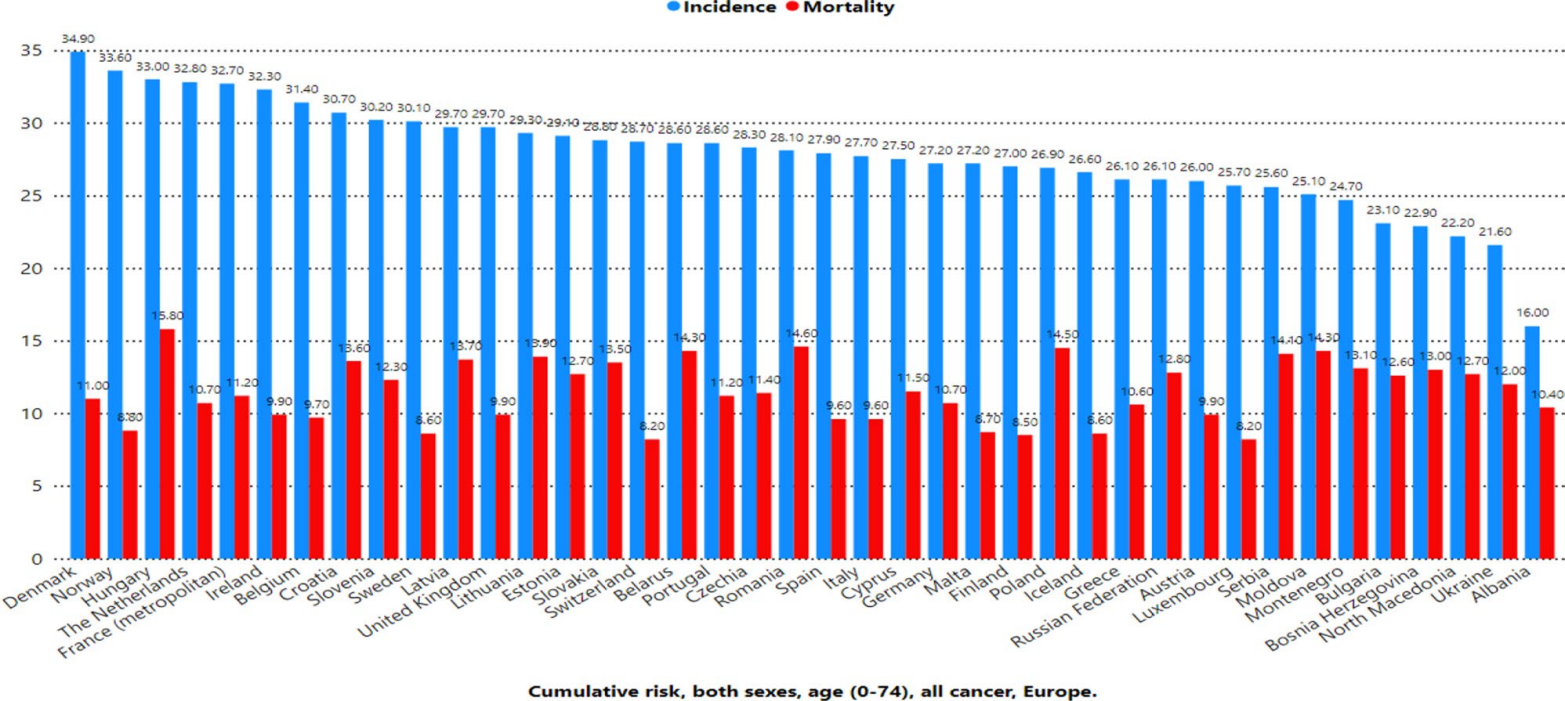
Estimated cumulative risk (%) incidence and mortality, both sexes, age (0–74) in 2022, all cancer, Europe

**Fig. 3:**
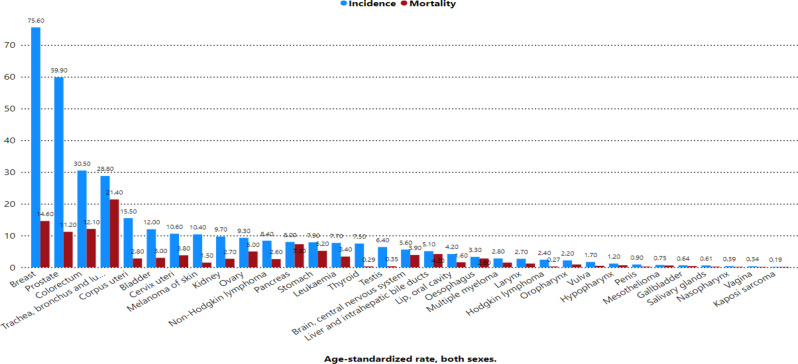
Age-standardized rate (world) per 100,000, incidence and mortality, both sexes, in 2022, all cancer, across Europe

**Fig. 4:**
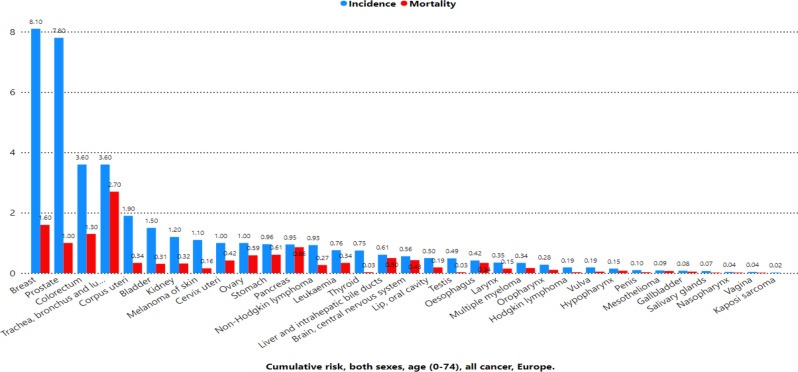
Estimated cumulative risk (%) incidence and mortality, both sexes, age (0–74) in 2022, all cancer, Europe

Figure 1 shows the age-standardized rates (ASRs) of cancer incidence and mortality across Europe. Denmark reported the highest incidence rate (374.7 per 100,000), followed by Norway (357.9), Ireland (344.7), and the Netherlands (341.4). In contrast, Albania had the lowest incidence rate (160.8), followed by Ukraine (199.9), North Macedonia (206.9), and Bosnia and Herzegovina (218.6). For mortality, Hungary recorded the highest rate (143.7 per 100,000), followed by Croatia (125.7), Lithuania (125.0), and Latvia (122.9). Conversely, the lowest mortality rates were observed in Switzerland (78.8), Luxembourg (77.3), and Iceland (79.8). Figure 2 highlights the cumulative risk of cancer incidence and mortality by age 75 across Europe. Denmark had the highest cumulative risk of developing cancer (34.9%), whereas Hungary had the highest risk of dying from cancer (15.8%). Figure 3 presents the age-standardized incidence and mortality rates (per 100,000) for various cancer types across Europe. Breast cancer had the highest incidence rate (70.6), followed by prostate cancer (48.7), Colorectum cancer (24.9), and lung cancer (24.7). In terms of mortality, lung cancer had the highest rate (17.7), followed by breast cancer (11.8), Colorectum cancer (8.6), and pancreatic cancer (5.6). Figure 4 shows the cumulative risks of incidence and mortality for different cancers. Breast cancer had the highest cumulative incidence risk (8.1%), whereas lung cancer had the highest cumulative mortality risk (2.7%). Cancers such as corpus uteri (14.3 incidence, 2.2 mortality) and bladder cancer (9.4 incidence, 1.8 mortality) presented moderate rates. Moreover, cancers such as Kaposi sarcoma (0.16 incidence, 0.02 mortality), gallbladder cancer (0.47 incidence, 0.29 mortality), and vaginal cancer (0.28 incidence, 0.09 mortality) had the lowest rates.

### Top 5 most frequent cancers in Europe by gender and region

**Fig. 5:**
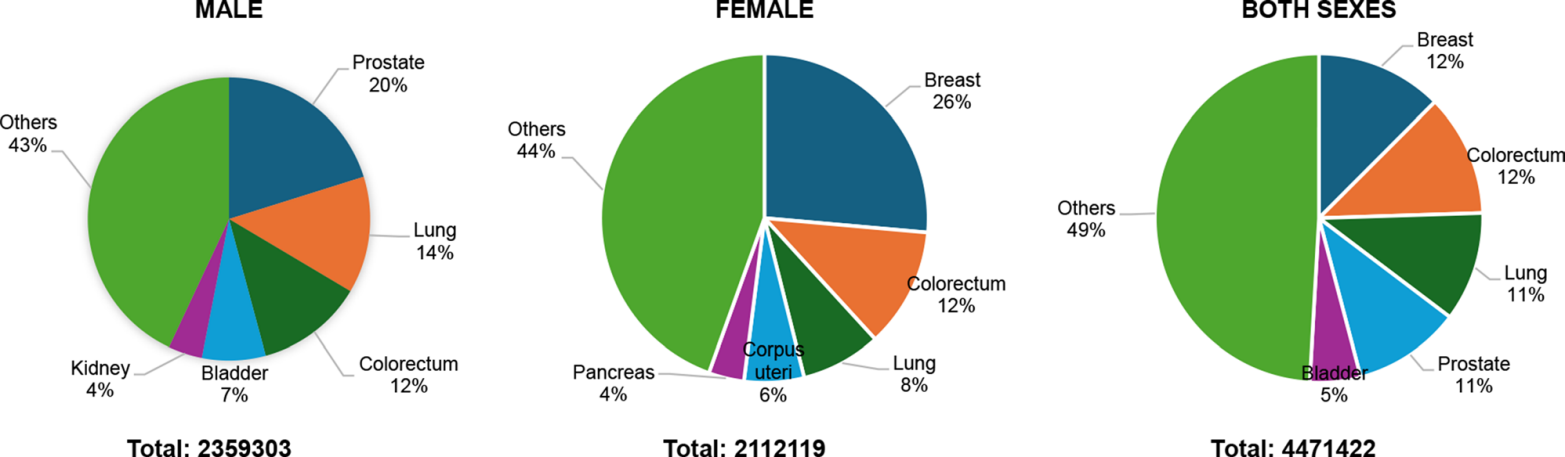
Top 5 most frequent cancers in 2022, all sexes, all over Europe

In 2022, Europe recorded 4,471,422 new cancer cases, with 2,359,303 cases in males and 2,112,119 in females Fig. 5. Among males, prostate cancer was the most common (20.0%, 473,011 cases), followed by lung cancer (13.4%, 317,070 cases), colorectal cancer (12.3%, 289,049 cases), bladder cancer (7.3%, 172,588 cases), and kidney cancer (3.9%, 91,652 cases). In females, breast cancer accounted for the highest proportion (26.4%, 557,532 cases), followed by colorectal cancer (11.8%, 249,213 cases), lung cancer (9.3%, 197,266 cases), corpus uteri cancer (5.9%, 124,874 cases), and pancreatic cancer (3.5%, 73,500 cases). When considering both sexes, breast cancer remained the most prevalent (12.5%), followed by colorectal (12.0%), lung (10.8%), prostate (10.6%), and bladder cancer (5.0%).

**Fig. 6:**
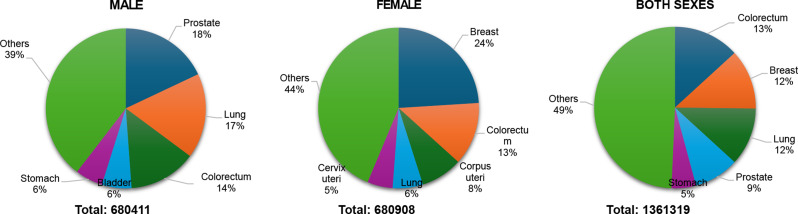
Top 5 most frequent cancers in Eastern Europe 2022, all sexes, all ages. Population(s) included: Belarus, Bulgaria, Czechia, Hungary, Moldova, Poland, Romania, Russian Federation, Slovakia, Ukraine

Eastern Europe reported 1,361,319 new cancer cases (680,411 in males, 680,908 in females) Fig. 6. Among males, prostate cancer (18.0%, 122,189 cases) was the most frequent, followed by lung (17.2%, 116,798 cases), colorectal (13.7%, 92,942 cases), bladder (6.0%, 40,678 cases), and stomach cancer (5.6%, 38,428 cases). In females, breast cancer (24.0%, 163,474 cases) dominated, followed by colorectal (12.7%, 86,657 cases), corpus uteri (8.4%, 57,095 cases), lung (6.1%, 41,349 cases), and cervical cancer (5.1%, 35,052 cases). When combining both sexes, colorectal cancer had the highest incidence (13.2%, 179,147 cases).

**Fig. 7:**
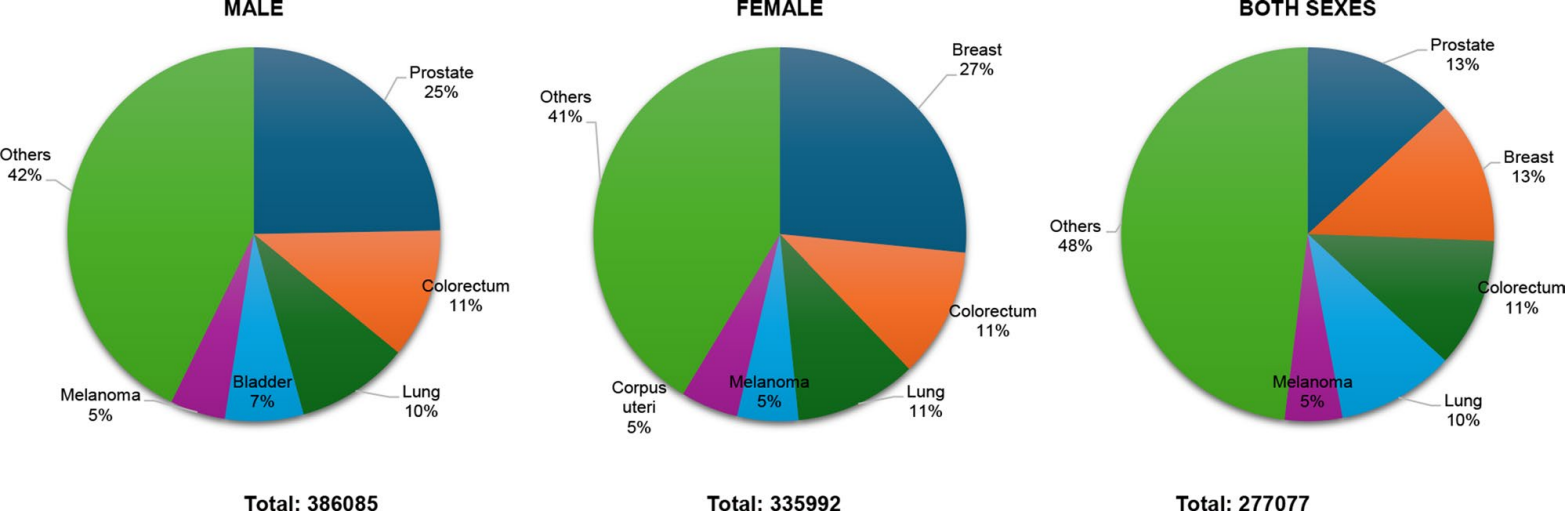
Top 5 most frequent cancers in Northern Europe 2022, all sexes, all ages. Population(s) included: Denmark, Estonia, Finland, Iceland, Ireland, Latvia, Lithuania, Norway, Sweden, United Kingdom, Faroe Islands, Aland Islands, Sark, Svalbard and Jan Mayen Islands, Guernsey, Jersey, Isle of Man

Northern Europe reported 722,077 new cases (386,085 in males, 335,992 in females). Prostate cancer (24.7%, 95,448 cases) was the leading cancer among males, followed by colorectal (11.2%, 43,220 cases), lung (9.8%, 37,931 cases), bladder (6.8%, 26,254 cases), and melanoma (4.8%, 18,365 cases). In females, breast cancer (26.6%, 89,324 cases) was the most common, followed by colorectal (11.2%, 37,704 cases), lung (10.6%, 35,677 cases), melanoma (5.3%, 17,761 cases), and corpus uteri cancer (5.0%, 16,736 cases). Prostate and breast cancer remained the most frequent overall Fig. 7.

**Fig. 8:**
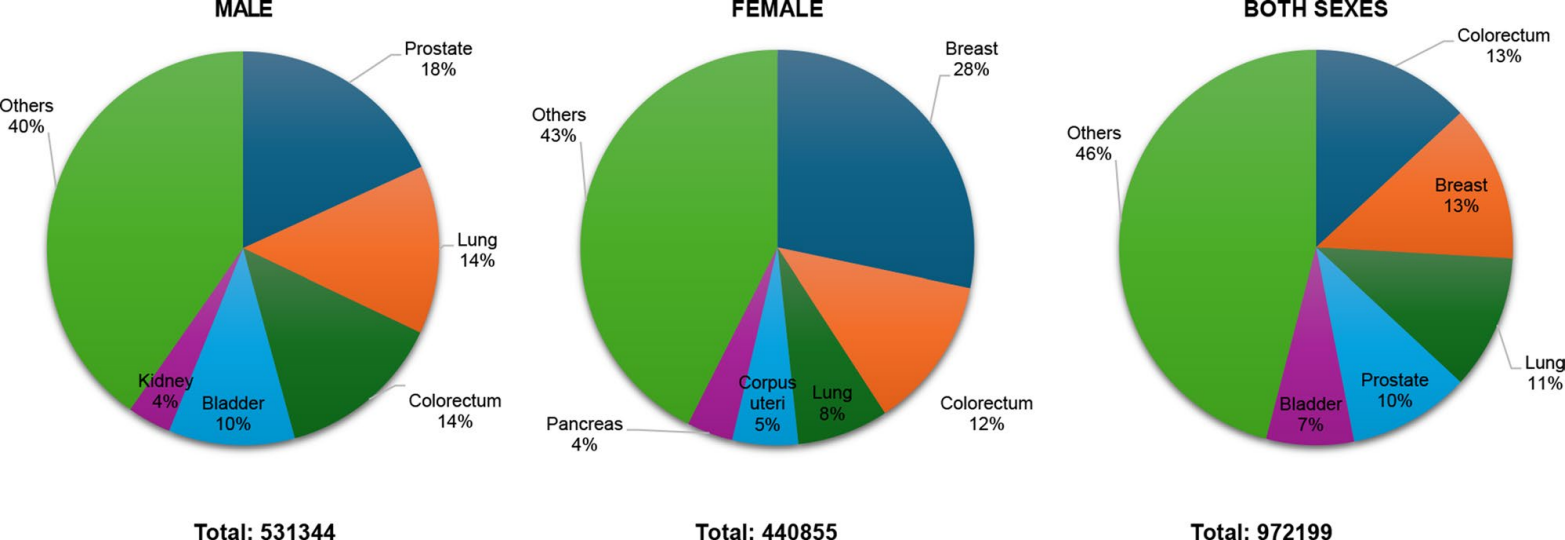
Top 5 most frequent cancers in Southern Europe 2022, all sexes, all ages. Population(s) included: Albania, Bosnia Herzegovina, Croatia, Cyprus, Greece, Italy, North Macedonia, Malta, Montenegro, Portugal, Serbia, Slovenia, Spain, Andorra, Gibraltar, Holy See*, San Marino

Southern Europe recorded 972,199 new cancer cases (531,344 in males, 440,855 in females) Fig. 8. Prostate cancer (18.2%, 96,952 cases) led among males, followed by lung (13.8%, 73,348 cases), colorectal (13.7%, 72,620 cases), bladder (10.4%, 55,523 cases), and kidney cancer (3.6%, 19,275 cases). Breast cancer was most frequent in females (28.3%, 124,621 cases), followed by colorectal (12.5%, 55,064 cases), lung (7.5%, 33,262 cases), corpus uteri (5.4%, 23,786 cases), and pancreatic cancer (3.8%, 16,712 cases). Colorectal cancer was the most common for both sexes (13.1%).

**Fig. 9:**
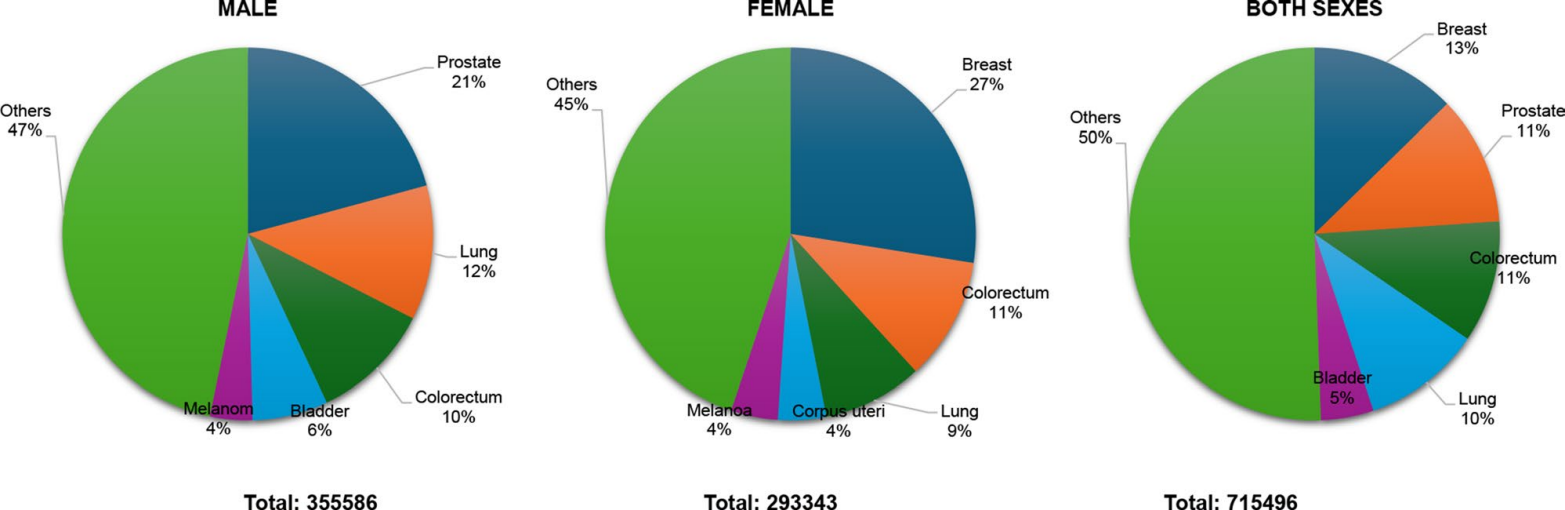
Top 5 most frequent cancers in Western Europe 2022, all sexes, all ages. Population(s) included: Austria, Belgium, France (metropolitan), Germany, Luxembourg, Switzerland, The Netherlands, Liechtenstein, Monaco

Western Europe reported 1,415,827 new cases (761,463 in males, 654,364 in females). Prostate cancer (20.8%, 158,422 cases) remained the most frequent in males, followed by lung (11.7%, 88,993 cases), colorectal (10.5%, 80,267 cases), bladder (6.6%, 50,133 cases), and melanoma (3.7%, 28,062 cases). In females, breast cancer (27.5%, 180,113 cases) was most prevalent, followed by colorectal (10.7%, 69,870 cases), lung (8.7%, 56,948 cases), corpus uteri (4.2%, 27,257 cases), and melanoma (4.1%, 26,833 cases). For both sexes, breast cancer had the highest overall incidence (12.7%), followed by prostate (11.2%), colorectal (10.6%), and lung cancer (10.3%) Fig. 9.

Cancer outcomes across European regions are significantly influenced by factors such as smoking prevalence and healthcare accessibility [37–39]. Smoking remains a major risk factor for various cancers, particularly lung cancer, with its prevalence varying markedly across Europe [40–42]. For instance, higher smoking rates in Central and Eastern Europe contribute to elevated lung cancer mortality in these regions [41]. Conversely, countries with robust tobacco control policies, like those in Northern and Western Europe, have experienced notable declines in smoking rates, leading to reduced cancer incidence and mortality [40, 41, 43, 44]. Healthcare accessibility further shapes cancer outcomes; nations with well-established healthcare systems and comprehensive cancer screening programs, such as those in Northern and Western Europe, often achieve earlier diagnoses and improved survival rates [45]. In contrast, regions with limited access to healthcare services face challenges, including delayed diagnoses and restricted treatment options, resulting in higher cancer-related mortality [42, 46]. Addressing these disparities through enhanced tobacco control measures and equitable healthcare access is essential for reducing regional differences in cancer burden across Europe [47].

## Discussion

This study provides a detailed analysis of cancer incidence and mortality across Europe, emphasizing significant regional disparities, sex differences, and trends in cancer occurrence and outcomes. In 2022, Europe recorded 4.47 million new cancer cases, a notable increase from the 4 million cases reported in 2021 [8]. This underscores the growing public health burden of cancer. Males presented higher age-standardized incidence rates (ASRs) than females did, which is consistent with global trends, where male populations generally face higher cancer risks [4, 48]. Prostate cancer was the most common type of cancer among males, whereas breast cancer dominated among females, reflecting established sex-specific cancer patterns [4, 49–53]. Northern Europe reported the highest cancer incidence rates for both sexes, with Denmark leading in ASR. This can be attributed to comprehensive screening programs [54–56], advanced detection methods, and heightened awareness. In contrast, Eastern Europe records lower incidence rates, likely reflecting limited healthcare investments [57], restricted access to screening programs [57, 58], and lifestyle factors [59]. These disparities highlight the critical need for equitable healthcare access and prevention strategies across Europe. The cancer mortality trends mirrored the incidence patterns, with Eastern Europe reporting the highest mortality rates, particularly among males. Challenges such as inadequate diagnostic tools, limited access to effective treatments, and insufficient palliative care services contribute to these elevated mortality rates. Hungary, which has Europe’s highest cancer mortality rate, underscores the urgent need to strengthen cancer care systems and implement effective prevention strategies, including tobacco and alcohol control measures [60, 61]. Conversely, Northern and Western Europe reported relatively lower mortality rates, reflecting stronger healthcare systems and comprehensive prevention programs [62]. The primary causes of cancer death were lung, Colorectum, and prostate cancers in males and breast, Colorectum, and lung cancers in females, which is consistent with global patterns [8, 63]. The leading role of lung cancer in mortality highlights the need for intensified smoking cessation campaigns and improved access to lung cancer screening, particularly for high-risk groups. Similarly, the high cumulative incidence of breast cancer (8.1%) calls for continued focus on early detection through mammography and awareness programs. Despite its lower incidence (0.95%), pancreatic cancer has a high mortality rate (0.86%). The cumulative risk of cancer incidence and mortality by age 75 revealed notable country-level differences. Denmark’s high cumulative risk of developing cancer (34.9%) reflects an advanced healthcare system that enables early diagnosis and accurate reporting. In contrast, Hungary’s high cumulative mortality risk (15.8%) underscores gaps in cancer treatment, care access, and palliative care services, necessitating urgent attention. The age-standardized rates of common cancers, such as breast and prostate cancers, align with established trends, whereas the relatively lower rates of rarer cancers, such as Kaposi sarcoma and vaginal cancer, point to the need for specialized interventions. The observed decline in rarer cancers, such as Kaposi sarcoma and vaginal cancer can be attributed to specific medical advancements and public health interventions. Kaposi sarcoma, which experienced a surge during the AIDS epidemic in the 1980s and early 1990s, has seen a significant reduction in incidence due to the widespread adoption of combination antiretroviral therapy (cART). This treatment effectively suppresses HIV replication, thereby restoring immune function and markedly decreasing the risk of Kaposi sarcoma among HIV-infected individuals [64].

Historically, Kaposi sarcoma was more prevalent among older adults of specific ethnic backgrounds, particularly those of Mediterranean or Eastern European descent. Excluding individuals over 75 years of age from analyses could, therefore, introduce an artifact, potentially underestimating the true incidence of this cancer in populations where it is traditionally more common [65]. The decrease in vaginal cancer incidence is largely associated with reduced exposure to known risk factors, notably persistent human papillomavirus (HPV) infection. The implementation of HPV vaccination programs has led to a substantial decline in infections with high-risk HPV types, which are implicated in the pathogenesis of vaginal and other anogenital cancers. Additionally, public health campaigns promoting smoking cessation have contributed to this decline, as smoking is a recognized cofactor in the development of HPV-related malignancies [66]. Furthermore, the historical use of Diethylstilbestrol (DES) for contraceptive purposes has been linked to an increased risk of vaginal clear cell adenocarcinoma in the daughters of women who used this drug during pregnancy. The discontinuation of DES use has corresponded with a subsequent decrease in these cases [67]. These observations underscore the importance of targeted medical treatments, effective vaccination programs, and public health policies in reducing the incidence of certain cancers. Ongoing efforts in these areas are essential to continuing this positive trend and addressing any emerging disparities in cancer outcomes.

While prostate, breast, colorectal, and lung cancer screening programs are critical for early detection, their availability and accessibility vary significantly across European countries. Disparities are more pronounced in Eastern and Southern Europe, where limited healthcare infrastructure, economic constraints, and lower public awareness hinder widespread implementation of systematic screening programs [68, 69]. For instance, organized breast cancer screening programs exist in most Western and Northern European countries, but participation rates remain low in regions like Eastern Europe due to financial barriers, lack of awareness, and insufficient healthcare resources [70]. Similarly, colorectal cancer screening programs are well-established in countries such as the Netherlands, the UK, and France, whereas some Eastern European countries still lack nationwide implementation [71]. Prostate cancer screening presents a unique challenge. While PSA testing was historically promoted as a screening tool, concerns about overdiagnosis and overtreatment have led to revised international guidelines favoring risk-based screening rather than universal PSA testing. Overdiagnosis can result in unnecessary interventions, including surgery and radiation, which may lead to complications such as incontinence and erectile dysfunction [72, 73]. The European Association of Urology now recommends targeted screening for high-risk groups, including men with a family history of prostate cancer or those of African descent, rather than blanket screening approaches [74].

Countries with comprehensive cancer prevention strategies, such as Finland and Sweden, have achieved significant reductions in colorectal and breast cancer mortality. For instance, Finland’s organized colorectal cancer screening program, utilizing fecal immunochemical tests (FIT), has been associated with decreased mortality rates [75–77]. Similarly, Sweden’s national breast cancer screening program has contributed to early detection and improved survival outcomes [78]. Conversely, regions with limited or underfunded screening initiatives, particularly in parts of Eastern and Southern Europe, experience higher mortality rates due to late-stage diagnoses and reduced access to early detection services [79].

Lifestyle interventions have also played a pivotal role in cancer prevention. Public health campaigns promoting smoking cessation and healthy dietary habits have led to declines in smoking-related cancers, such as lung cancer, and diet-related cancers, including colorectal cancer. For example, the Felix Burda Foundation in Germany has been instrumental in raising awareness about colorectal cancer and advocating for early screening, resulting in increased participation rates and early-stage detections [80]. Additionally, studies suggest that engaging in physical activity during the morning and evening could reduce the risk of colorectal cancer by 11%, highlighting the importance of promoting regular exercise as a preventive measure [81, 82].

Emerging cancer types, such as those associated with human papillomavirus (HPV) and hepatitis infections, are expected to increase in prevalence across Europe. HPV-related cancers, including cervical, oropharyngeal, and anal cancers, may rise in countries with low vaccination uptake, particularly in Eastern Europe, where HPV vaccination programs are not as widespread [83, 84]. Similarly, the burden of liver cancer, associated with chronic hepatitis B and C infections, is projected to increase in regions with inadequate vaccination and screening programs [85, 86].

Addressing cancer disparities across Europe requires more than just advocating for better programs; it necessitates targeted policy interventions that consider regional healthcare capacities, economic constraints, and cultural factors. Tailored strategies, such as financial incentives for screening participation, public awareness campaigns, and infrastructure improvements, can help bridge the gap in cancer prevention, particularly in regions like Eastern and Southern Europe, where barriers to healthcare access persist [27]. Governments and health organizations should focus on reducing healthcare inequalities by implementing evidence-based prevention programs, including smoking cessation, obesity reduction, and expanded cancer screening, which are critical for reducing the cancer burden [87]. Prostate, breast, colorectal, and lung cancer screening programs should be widely accessible, as early detection significantly reduces mortality rates [88]. Moreover, to address these disparities and emerging trends, it is essential to enhance cancer prevention strategies across Europe, which includes strengthening vaccination programs, expanding access to screening and early detection services, and promoting lifestyle modifications to reduce cancer risk [89]. Implementing evidence-based policies and region-specific interventions will be crucial in reducing the cancer burden and achieving equity in cancer care across the continent.

## Conclusion

In conclusion, this study underscores significant disparities in cancer incidence and mortality across Europe, driven by regional healthcare inequalities, gender-specific patterns, and variations in prevention and early detection programs.

Western and Northern European countries, which generally report lower mortality rates despite high incidence rates, tend to have well-established cancer prevention and early detection programs, higher healthcare expenditures, and broader access to advanced treatment options. In contrast, Eastern and Southern European countries often exhibit higher mortality-to-incidence ratios, reflecting challenges such as limited access to screening, delayed diagnoses, and disparities in cancer care quality. A more detailed examination of specific cancers, such as lung and colorectal cancer, further illustrates these disparities. Lung cancer mortality remains highest in Eastern Europe, where smoking prevalence is significantly higher than in Western Europe, despite similar incidence rates. Meanwhile, colorectal cancer screening programs have led to declining mortality rates in countries like the Netherlands and the UK, whereas limited screening availability in parts of Eastern Europe continues to result in poorer outcomes.

These findings highlight the urgent need for tailored public health strategies, particularly in regions with limited healthcare access, to improve cancer prevention, early diagnosis, and treatment. Targeted interventions, such as expanded cancer screening programs, effective tobacco and alcohol control, and strengthened healthcare systems, have the potential to reduce the cancer burden and improve outcomes across Europe. Addressing these challenges through evidence-based policies and region-specific interventions is critical to achieving equity in cancer care and advancing public health across the continent.

### Limitation

This study has several limitations that should be acknowledged. First, the reliance on GLOBOCAN 2022 data introduces potential estimation bias, as cancer incidence sources vary across Europe. While Northern European countries use high-quality national cancer registries, many Eastern and Southern European countries rely on modelled estimates derived from mortality-to-incidence ratios, which may not accurately reflect true incidence patterns. Additionally, in cases where country-specific cancer registries are unavailable, data from neighbouring countries are used, potentially leading to misclassification and inaccuracies due to differences in healthcare systems, screening programs, and population demographics. Second, the study is based on cross-sectional data from 2022, which does not account for longitudinal trends in cancer incidence and mortality that may be influenced by changes in screening, diagnosis, and treatment over time. Third, the analysis is conducted at the population level, limiting the ability to assess individual-level risk factors, socioeconomic disparities, and healthcare access issues that contribute to variations in cancer burden. The study also does not consider subnational differences, which can be significant, particularly between urban and rural areas where cancer prevention and treatment access may vary. Furthermore, the study does not examine modifiable risk factors such as tobacco use, alcohol consumption, diet, and environmental exposures, which are crucial for understanding cancer patterns and guiding prevention strategies. Another limitation is the absence of cancer survival data, stage at diagnosis, and treatment outcomes, which restricts the study’s ability to assess the effectiveness of healthcare systems in managing cancer. Finally, while the study follows ethical guidelines for secondary data use, it lacks direct data validation, making it difficult to assess potential biases in reporting, registry coverage, or missing data.

## Data Availability

The data used in this study are available to the public from the Global Cancer Observatory’s Cancer Today (GLOBOCAN): https://gco.iarc.who.int/today/en/dataviz.

## Abbreviations

(AIDS): Acquired Immunodeficiency Syndrome
(cART): Adoption of Combination Antiretroviral Therapy
(ASRs): Age-Standardized Rates
(DES): Diethylstilbestrol
(ECIR): European Cancer Inequalities Registry
(EU): European Union
(FIT): Fecal Immunochemical Test
(GCO): Global Cancer Observatory
(HPV): Human Papillomavirus
(IARC): International Agency for Research on Cancer
(ICD-10): International Classification of Diseases, Tenth Edition
(PBCRs): Population-Based Cancer Registries
(PSA): Prostate-Specific Antigen
(UK): United Kingdom

## References

[1] Tran KB, Lang JJ, Compton K, Xu R, Acheson AR, Henrikson HJ, et al. The global burden of cancer attributable to risk factors, 2010–19: a systematic analysis for the global burden of disease study 2019. Lancet. 2022;400(10352):563–91. 10.1016/S0140-6736(22)01438-6. 35988567 10.1016/S0140-6736(22)01438-6 PMC9395583

[2] Shariat SF. A pan-European total cancer prevalence canvas: a benchmark for advancing strategic interventions. Lancet Oncol. 2024;25(3):266–7. 10.1016/S1470-2045(24)00089-5. 38423039 10.1016/S1470-2045(24)00089-5

[3] Bencina G, Sabale U, Morais E, Ovcinnikova O, Oliver E, Shoel H, et al. Burden and indirect cost of vaccine-preventable cancer mortality in Europe. J Med Econ. 2024;27(sup2):30–40. 10.1080/13696998.2024.2374684. 39010684 10.1080/13696998.2024.2374684

[4] Bray F, Laversanne M, Sung H, Ferlay J, Siegel RL, Soerjomataram I, et al. Global cancer statistics 2022: GLOBOCAN estimates of incidence and mortality worldwide for 36 cancers in 185 countries. Cancer J Clin. 2022;74(3):229–63. 10.3322/caac.21834. 10.3322/caac.21834 38572751

[5] WHO. Latest global cancer data: Cancer burden rises to 18.1 million new cases and 9.6 million cancer deaths in 2018: International Agency for Research on Cancer. 2018 [263:[Available from: https://www.iarc.who.int/wp-content/uploads/2018/09/pr263_E.pdf

[6] Charalambous A, Price R, Jha P. Accelerating progress on EU cancer control. Lancet Oncol. 2024;25(2):158–60. 10.1016/S1470-2045(24)00002-0. https://doi.org/https://. 38301686 10.1016/S1470-2045(24)00002-0

[7] Li L, Shan T, Zhang D, Ma F. Nowcasting and forecasting global aging and cancer burden: analysis of data from the GLOBOCAN and global burden of disease study. J Natl Cancer Cent. 2024;4(3):223–32. 10.1016/j.jncc.2024.05.002. 39281725 10.1016/j.jncc.2024.05.002 PMC11401500

[8] Dyba T, Randi G, Bray F, Martos C, Giusti F, Nicholson N, et al. The European cancer burden in 2020: incidence and mortality estimates for 40 countries and 25 major cancers. Eur J Cancer. 2021;157:308–47. 10.1016/j.ejca.2021.07.039. 34560371 10.1016/j.ejca.2021.07.039 PMC8568058

[9] Ju W, Zheng R, Wang S, Zhang S, Zeng H, Chen R, et al. The occurrence of cancer in ageing populations at global and regional levels, 1990 to 2019. Age Ageing. 2023;52(9). 10.1093/ageing/afad043. 10.1093/ageing/afad043 37725972

[10] Fan Y, Jiang Y, Gong L, Wang Y, Su Z, Li X, et al. Epidemiological and demographic drivers of lung cancer mortality from 1990 to 2019: results from the global burden of disease study 2019. Front Public Health. 2023;11. 10.3389/fpubh.2023.1054200. 10.3389/fpubh.2023.1054200 PMC10196253 37213644

[11] Newman L. Oncologic anthropology: global variations in breast cancer risk, biology, and outcome. J Surg Oncol. 2023;128(6):959–66. 10.1002/jso.27459. https://doi.org/https://doi.org/. 37814598 10.1002/jso.27459

[12] Sedeta ET, Jobre B, Avezbakiyev B. Breast cancer: global patterns of incidence, mortality, and trends. J Clin Oncol. 2023;41(16suppl):10528. 10.1200/JCO.2023.41.16_suppl.10528.

[13] Yu S, Cai X, Wang X, Lin X, Cai S. Disease burden of breast cancer and risk factors in Europe 44 countries, 1990–2019: findings of the global burden of disease study 2019. Front Endocrinol. 2024;15. 10.3389/fendo.2024.1405204. 10.3389/fendo.2024.1405204 PMC11153740 38846496

[14] OECD. Beating Cancer Inequalities in the EU2024.

[15] Amato O, Guarneri V, Girardi F. Epidemiology trends and progress in breast cancer survival: earlier diagnosis, new therapeutics. Curr Opin Oncol. 2023;35(6):612–9. 10.1097/cco.0000000000000991. 37681462 10.1097/CCO.0000000000000991 PMC10566595

[16] Kashyap D, Pal D, Sharma R, Garg VK, Goel N, Koundal D, et al. Global increase in breast Cancer incidence: risk factors and preventive measures. Biomed Res Int. 2022;2022:9605439. 10.1155/2022/9605439. 35480139 10.1155/2022/9605439 PMC9038417

[17] Henderson RH, French D, Maughan T, Adams R, Allemani C, Minicozzi P, et al. The economic burden of colorectal cancer across Europe: a population-based cost-of-illness study. Lancet Gastroenterol Hepatol. 2021;6(9):709–22. 10.1016/S2468-1253(21)00147-3. 34329626 10.1016/S2468-1253(21)00147-3

[18] Falcaro M, Soldan K, Ndlela B, Sasieni P. Effect of the HPV vaccination programme on incidence of cervical cancer and grade 3 cervical intraepithelial neoplasia by socioeconomic deprivation in England: population based observational study. BMJ. 2024;385:e077341. 10.1136/bmj-2023-077341. 38749552 10.1136/bmj-2023-077341 PMC11094700

[19] Ferraris G, Coppini V, Monzani D, Grasso R, Kirac I, Horgan D, et al. Addressing disparities in European cancer outcomes: a qualitative study protocol of the BEACON project. Front Psychol. 2024;15:1252832. 10.3389/fpsyg.2024.1252832. 38469221 10.3389/fpsyg.2024.1252832 PMC10925749

[20] Ahmad I, Jasim SA, Sharma MK, Hjazi SRJ, Mohammed A. New paradigms to break barriers in early cancer detection for improved prognosis and treatment outcomes. J Gene Med. 2024;26(8):e3730. 10.1002/jgm.3730. 39152771 10.1002/jgm.3730

[21] Noor J, Chaudhry A, Batool S. Microfluidic technology, artificial intelligence, and biosensors as advanced technologies in Cancer screening: A review Article. Cureus. 2023;15(5):e39634. 10.7759/cureus.39634. 37388583 10.7759/cureus.39634 PMC10305590

[22] Annette L. Fighting the good fight: Europe’s Beating Cancer Plan. Impact. 2023;2023(3):4–5 10.21820/23987073.2023.3.4.

[23] Corso G, Janssens JP, La Vecchia C. Cancer prevention: innovative strategies in the role of the European Cancer prevention organization. Eur J Cancer Prev. 2023;32(3):203–6. 10.1097/cej.0000000000000782. 36719848 10.1097/CEJ.0000000000000782

[24] Ferlay JEM, Lam F, Laversanne M, Colombet M, Mery L, Piñeros M, Znaor A, Soerjomataram I, Bray F. Global Cancer Observatory: Cancer Today: Lyon, France: International Agency for Research on Cancer; 2024 [cited 2025 15 February]. Available from: https://gco.iarc.who.int/today/en/data-sources-methods

[25] Gehrmann J, Beyan O. Stud Health Technol Inf. 2024;316:9–13. 10.3233/shti240332. Data Quality in Medical Real-World Data - An Oncological Use Case. 10.3233/SHTI240332 39176661

[26] Martos C, Giusti F, Van Eycken L, Visser O, Editorial. Joining efforts to improve data quality and harmonization among European population-based cancer registries. Front Oncol. 2024;14. 10.3389/fonc.2024.1496574. 10.3389/fonc.2024.1496574 PMC11496113 39445064

[27] Giusti F, Martos C, Carvalho RN, Zadnik V, Visser O, Bettio M, et al. Facing further challenges in cancer data quality and harmonisation. Front Oncol. 2024;14. 10.3389/fonc.2024.1438805. 10.3389/fonc.2024.1438805 PMC11307262 39119089

[28] Stepien M, Rodriguez Rasero F, Ben E, Nicholl C. Monitoring inequalities in cancer prevention and care in Europe. Eur J Pub Health. 2023;33. 10.1093/eurpub/ckad160.1679. Supplement_2.

[29] Asensio M, Amaral E. Disparities in cancer outcomes: A comprehensive analysis of cancer incidence, mortality, and prevalence in Europe. GHES. 2024;2(2). 10.36922/ghes.3216.

[30] Zheng R, Wang S, Zhang S, Zeng H, Chen R, Sun K, et al. Global, regional, and National lifetime probabilities of developing cancer in 2020. Sci Bull (Beijing). 2023;68(21):2620–8. 10.1016/j.scib.2023.09.041. 37821267 10.1016/j.scib.2023.09.041 PMC10640926

[31] McNutt L-A, Krug. Allison. cumulative incidence. Encyclopedia Britannica, 2016 [cited 2025 16 February]. Available from: https://www.britannica.com/science/cumulative-incidence.

[32] Xiang D, Hu S, Mai T, Zhang X, Zhang L, Wang S, et al. Worldwide cancer statistics of adults over 75 years old in 2019: a systematic analysis of the global burden of disease study 2019. BMC Public Health. 2022;22(1):1979. 10.1186/s12889-022-14412-1. 36307792 10.1186/s12889-022-14412-1 PMC9617321

[33] Bizuayehu HM, Ahmed KY, Kibret GD, Dadi AF, Belachew SA, Bagade T, et al. Global disparities of Cancer and its projected burden in 2050. JAMA Netw Open. 2024;7(11):e2443198–e. 10.1001/jamanetworkopen.2024.43198. 39499513 10.1001/jamanetworkopen.2024.43198 PMC11539015

[34] Sasieni PD, Shelton J, Ormiston-Smith N, Thomson CS, Silcocks PB. What is The lifetime risk of developing cancer? The effect of adjusting for multiple primaries. Br J Cancer. 2011;105(3):460–5. 10.1038/bjc.2011.250. 21772332 10.1038/bjc.2011.250 PMC3172907

[35] White MC, Holman DM, Boehm JE, Peipins LA, Grossman M, Henley SJ. Age and cancer risk: a potentially modifiable relationship. Am J Prev Med. 2014;46(3 Suppl 1):S7–15. 10.1016/j.amepre.2013.10.029. 24512933 10.1016/j.amepre.2013.10.029 PMC4544764

[36] Nelson C. Some Older Adults Find an Age Cutoff For Colon Cancer Screenings ‘Unacceptable’—Here’s Why Doctors Say It Exists 2024 [cited 2025 16 February]. Available from: https://www.health.com/colorectal-cancer-screening-age-cap-survey-8761413?utm_source=chatgpt.com

[37] OECD. Beating Cancer inequalities in the EU: spotlight on Cancer prevention and early detection, OECD health policy studies. Paris: OECD Publishing; 2024.

[38] Gerwin Winter EL. Tackling disparities in cancer care across Europe: how can we improve access for all patients in Europe? 2023 [cited 2025 17 February]. Available from: https://www.oncology-central.com/tackling-disparities-in-cancer-care-across-europe-how-can-we-improve-access-for-all-patients-in-europe/

[39] Ciuba A, Wnuk K, Nitsch-Osuch A, Kulpa M. Health care accessibility and breast Cancer mortality in Europe. Int J Environ Res Public Health. 2022;19(20). 10.3390/ijerph192013605. 10.3390/ijerph192013605 PMC9602737 36294189

[40] Teshima A, Laverty AA, Filippidis FT. Burden of current and past smoking across 28 European countries in 2017: A cross-sectional analysis. Tob Induc Dis. 2022;20(June):1–11. 10.18332/tid/149477. 10.18332/tid/149477 PMC9194927 35799620

[41] Long D, Mackenbach J, Martikainen P, Lundberg O, Brønnum-Hansen H, Bopp M, et al. Smoking and inequalities in mortality in 11 European countries: a birth cohort analysis. Popul Health Metrics. 2021;19(1):3. 10.1186/s12963-021-00247-2. 10.1186/s12963-021-00247-2 PMC7847590 33516235

[42] Islami F, Torre LA, Jemal A. Global trends of lung cancer mortality and smoking prevalence. Translational Lung Cancer Res. 2015;4(4):327–38. 10.3978/j.issn.2218-6751.2015.08.04 PMC4549470 26380174

[43] Islami F, Torre LA, Jemal A. Global trends of lung cancer mortality and smoking prevalence. Transl Lung Cancer Res. 2015;4(4):327–38. 10.3978/j.issn.2218-6751.2015.08.04. 26380174 10.3978/j.issn.2218-6751.2015.08.04 PMC4549470

[44] Ten Haaf K. Confronting the burden of tobacco-related lung cancer in Europe in the next decades. Lancet Reg Health Eur. 2021;4:100085. 10.1016/j.lanepe.2021.100085. 34557813 10.1016/j.lanepe.2021.100085 PMC8454878

[45] organization Ec. Equal access to affordable and optimal cancer care, including the right to a second opinion. [cited 2025 17 February]. Available from: https://www.europeancancer.org/content/the-code-equal-access.html

[46] Borràs JM, Fernandez E, Gonzalez JR, Negri E, Lucchini F, La Vecchia C, et al. Lung cancer mortality in European regions (1955–1997). Annals of Oncology. 2003;14(1):159– 61 10.1093/annonc/mdg016. 10.1093/annonc/mdg016 12488308

[47] WHO. Tobacco-related cancers and prevention 2020 [cited 2025 17 February]. Available from: https://cancerpreventioneurope.iarc.fr/european-code-against-cancer/tobacco-related-cancers-and-prevention/

[48] Desai K, Baralo B, Kulkarni A, Keshava VE, Iqbal S, Ali H, et al. Cancer statistics: the united States vs. worldwide. J Clin Oncol. 2024;42(16suppl):e23276–e. 10.1200/JCO.2024.42.16_suppl.e23276.

[49] Haider M, Lange PH. Chapter 19 - Breast and Prostate Cancers: A Comparison of Two Endocrinologic Malignancies. In: Mydlo JH, Godec CJ, editors. Prostate Cancer (Second Edition). San Diego: Academic Press; 2016. pp. 157– 65.

[50] López-Abente G, Mispireta S, Pollán M. Breast and prostate cancer: an analysis of common epidemiological features in mortality trends in Spain. BMC Cancer. 2014;14(1):874. 10.1186/1471-2407-14-874. 25421124 10.1186/1471-2407-14-874 PMC4251688

[51] De Silva F, Alcorn J. A Tale of two cancers: A current concise overview of breast and prostate Cancer. Cancers. 2022;14(12):2954. 35740617 10.3390/cancers14122954 PMC9220807

[52] Sung H, Ferlay J, Siegel RL, Laversanne M, Soerjomataram I, Jemal A, et al. Global Cancer statistics 2020: GLOBOCAN estimates of incidence and mortality worldwide for 36 cancers in 185 countries. CA: A Cancer. J Clin. 2021;71(3):209–49. 10.3322/caac.21660. 10.3322/caac.21660 33538338

[53] Chen Z, Xu L, Shi W, Zeng F, Zhuo R, Hao X, et al. Trends of female and male breast cancer incidence at the global, regional, and National levels, 1990–2017. Breast Cancer Res Treat. 2020;180(2):481–90. 10.1007/s10549-020-05561-1. 32056055 10.1007/s10549-020-05561-1

[54] Beauté J, Innocenti F. Differences between males and females in infectious diseases notifications in the EU/EEA, 2012 to 2021. Eurosurveillance. 2024;29(33):2300655. 10.2807/1560-7917.ES.2024.29.33.2300655. 39149823 10.2807/1560-7917.ES.2024.29.33.2300655 PMC11328500

[55] Milutinovic S, Escarcega RO, Lopez-Mattei J, Petrovic M. Gender disparities and infective endocarditis burden in the united States and European union: a comparative analysis. Eur Heart J. 2024;45. 10.1093/eurheartj/ehae666.2618. Supplement_1.

[56] Jain A, Jani C, Patel S, Raval M, Singh H. Age-standardized incidence, mortality rates, mortality-to-incidence ratios, and disability-adjusted life years for acute lymphoblastic leukemia in the European union 15 + countries, Australia, and united States of America. J Clin Oncol. 2023;41(16suppl):e19052–e. 10.1200/JCO.2023.41.16_suppl.e19052.

[57] Vrdoljak E, Bodoky G, Jassem J, Popescu R, Pirker R, Čufer T, et al. Expenditures on oncology drugs and Cancer Mortality-to‐Incidence ratio in central and Eastern Europe. Oncologist. 2018;24(1):e30–7. 10.1634/theoncologist.2018-0093. 30181313 10.1634/theoncologist.2018-0093 PMC6324644

[58] Trojanowski M, Radomyski P, Kycler W, Michalek IM. Decrease in the number of new cancer diagnoses during the first year of the COVID-19 pandemic– cohort study of 3.5 million individuals in Western Poland. Front Oncol. 2023;13. 10.3389/fonc.2023.1230289. 10.3389/fonc.2023.1230289 PMC10765942 38179170

[59] Santucci C, Patel L, Malvezzi M, Wojtyla C, La Vecchia C, Negri E, et al. Persisting cancer mortality gap between Western and Eastern Europe. Eur J Cancer. 2022;165:1–12. 10.1016/j.ejca.2022.01.007. 35189536 10.1016/j.ejca.2022.01.007

[60] Coppini V, Ferraris G, Ferrari MV, Dahò M, Kirac I, Renko I, et al. Patients’ perspectives on cancer care disparities in central and Eastern European countries: experiencing taboos, misinformation and barriers in the healthcare system. Front Oncol. 2024;14:1420178. 10.3389/fonc.2024.1420178. 39184044 10.3389/fonc.2024.1420178 PMC11341380

[61] Kenessey I, Nagy P, Polgár C. [The Hungarian situation of cancer epidemiology in the second decade of the 21st century]. Magy Onkol. 2022;66(3):175–84. 36200497

[62] Vrdoljak E, Bodoky G, Jassem J, Popescu RA, Mardiak J, Pirker R, et al. Cancer control in central and Eastern Europe: current situation and recommendations for improvement. Oncologist. 2016;21(10):1183–90. 10.1634/theoncologist.2016-0137. 27401890 10.1634/theoncologist.2016-0137 PMC5061531

[63] Bray F, Laversanne M, Sung H, Ferlay J, Siegel RL, Soerjomataram I, et al. Global cancer statistics 2022: GLOBOCAN estimates of incidence and mortality worldwide for 36 cancers in 185 countries. CA Cancer J Clin. 2024;74(3):229–63. 10.3322/caac.21834. 38572751 10.3322/caac.21834

[64] Lodi S, Guiguet M, Costagliola D, Fisher M, de Luca A, Porter K. Kaposi sarcoma incidence and survival among HIV-infected homosexual men after HIV seroconversion. J Natl Cancer Inst. 2010;102(11):784–92. 10.1093/jnci/djq134. 20442214 10.1093/jnci/djq134 PMC2879418

[65] Liu Z, Fang Q, Zuo J, Minhas V, Wood C, Zhang T. The world-wide incidence of Kaposi’s sarcoma in the HIV/AIDS era. HIV Med. 2018;19(5):355–64. 10.1111/hiv.12584. 29368388 10.1111/hiv.12584

[66] UK. CR. Risks and causes of vaginal cancer 2022 [cited 2025 17 February]. Available from: https://www.cancerresearchuk.org/about-cancer/vaginal-cancer/risk-causes?utm_source=chatgpt.com

[67] Wu X, Matanoski G, Chen VW, Saraiya M, Coughlin SS, King JB, et al. Descriptive epidemiology of vaginal cancer incidence and survival by race, ethnicity, and age in the united States. Cancer. 2008;113(S10):2873–82. 10.1002/cncr.23757. 18980291 10.1002/cncr.23757

[68] Arnold M, Rutherford MJ, Bardot A, Ferlay J, Andersson TM, Myklebust T, et al. Progress in cancer survival, mortality, and incidence in seven high-income countries 1995–2014 (ICBP SURVMARK-2): a population-based study. Lancet Oncol. 2019;20(11):1493–505. 10.1016/s1470-2045(19)30456-5. 31521509 10.1016/S1470-2045(19)30456-5 PMC6838671

[69] Maringe C, Spicer J, Morris M, Purushotham A, Nolte E, Sullivan R, et al. The impact of the COVID-19 pandemic on cancer deaths due to delays in diagnosis in England, UK: a National, population-based, modelling study. Lancet Oncol. 2020;21(8):1023–34. 10.1016/s1470-2045(20)30388-0. 32702310 10.1016/S1470-2045(20)30388-0 PMC7417808

[70] Pashayan N, Antoniou AC, Ivanus U, Esserman LJ, Easton DF, French D, et al. Personalized early detection and prevention of breast cancer: ENVISION consensus statement. Nat Rev Clin Oncol. 2020;17(11):687–705. 10.1038/s41571-020-0388-9. 32555420 10.1038/s41571-020-0388-9 PMC7567644

[71] Cardoso R, Guo F, Heisser T, Hoffmeister M, Brenner H. Utilisation of colorectal Cancer screening tests in European countries by type of screening offer: results from the European health interview survey. Cancers. 2020;12(6):1409. 32486077 10.3390/cancers12061409 PMC7352919

[72] Schröder FH, Hugosson J, Roobol MJ, Tammela TL, Ciatto S, Nelen V, et al. Screening and prostate-cancer mortality in a randomized European study. N Engl J Med. 2009;360(13):1320–8. 10.1056/NEJMoa0810084. 19297566 10.1056/NEJMoa0810084

[73] Hugosson J, Roobol MJ, Månsson M, Tammela TLJ, Zappa M, Nelen V, et al. A 16-yr Follow-up of the European randomized study of screening for prostate Cancer. Eur Urol. 2019;76(1):43–51. 10.1016/j.eururo.2019.02.009. 30824296 10.1016/j.eururo.2019.02.009 PMC7513694

[74] Mottet N, van den Bergh RCN, Briers E, Van den Broeck T, Cumberbatch MG, De Santis M, et al. EAU-EANM-ESTRO-ESUR-SIOG guidelines on prostate Cancer-2020 update. Part 1: screening, diagnosis, and local treatment with curative intent. Eur Urol. 2021;79(2):243–62. 10.1016/j.eururo.2020.09.042. 33172724 10.1016/j.eururo.2020.09.042

[75] Relander P, Rauhaniemi E, Löyttyniemi E, Salminen K, Carpelan A, Koffert J. First local results of the Finnish FIT-based colorectal cancer screening program - high yield, low complications. Scand J Gastroenterol. 2025;1–6. 10.1080/00365521.2025.2458062. 10.1080/00365521.2025.2458062 39893517

[76] Ola I, Cardoso R, Hoffmeister M, Brenner H. Utilization of colorectal cancer screening tests across European countries: a cross-sectional analysis of the European health interview survey 2018–2020. Lancet Reg Health Eur. 2024;41:100920. 10.1016/j.lanepe.2024.100920. 38707865 10.1016/j.lanepe.2024.100920 PMC11067466

[77] Santucci C, Mignozzi S, Malvezzi M, Boffetta P, Collatuzzo G, Levi F, et al. European cancer mortality predictions for the year 2024 with focus on colorectal cancer. Ann Oncol. 2024;35(3):308–16. 10.1016/j.annonc.2023.12.003. 38286716 10.1016/j.annonc.2023.12.003

[78] Roginski M, Sifaki-Pistolla D, Stomby A, Velivasaki G, Faresjö T, Lionis C, et al. Paradoxes of breast cancer incidence and mortality in two corners of Europe. BMC Cancer. 2022;22(1):1123. 10.1186/s12885-022-10243-w. 36319987 10.1186/s12885-022-10243-w PMC9628067

[79] Guthmuller S, Carrieri V, Wübker A. Effects of organized screening programs on breast cancer screening, incidence, and mortality in Europe. J Health Econ. 2023;92:102803. 10.1016/j.jhealeco.2023.102803. 37688931 10.1016/j.jhealeco.2023.102803

[80] Foundation FB. Successful prevention 2024 [cited 2025 17 February]. Available from: https://www.burda.com/en/company/responsibility/foundations/felix-burda-foundation/

[81] Stein MJ, Baurecht H, Bohmann P, Fervers B, Fontvieille E, Freisling H, et al. Diurnal timing of physical activity and risk of colorectal cancer in the UK biobank. BMC Med. 2024;22(1):399. 10.1186/s12916-024-03632-4. 39289682 10.1186/s12916-024-03632-4 PMC11409794

[82] Guardian T. Early morning and evening activity could ‘reduce bowel cancer risk by 11% 2024 [cited 2025 17 February]. Available from: https://www.theguardian.com/society/2024/nov/06/early-morning-and-evening-activity-could-reduce-bowel-cancer-risk

[83] Villain P, Gonzalez P, Almonte M, Franceschi S, Dillner J, Anttila A, et al. European code against Cancer 4th edition: infections and Cancer. Cancer Epidemiol. 2015;39. 10.1016/j.canep.2015.10.006. S120-S38. 10.1016/j.canep.2015.10.006 26589774

[84] Branda F, Pavia G, Ciccozzi A, Quirino A, Marascio N, Gigliotti S, et al. Human papillomavirus (HPV) vaccination: progress, challenges, and future directions in global immunization strategies. Vaccines. 2024;12(11):1293. 39591195 10.3390/vaccines12111293 PMC11598998

[85] Gnyawali B, Pusateri A, Nickerson A, Jalil S, Mumtaz K. Epidemiologic and socioeconomic factors impacting hepatitis B virus and related hepatocellular carcinoma. World J Gastroenterol. 2022;28(29):3793–802. 10.3748/wjg.v28.i29.3793. 36157533 10.3748/wjg.v28.i29.3793 PMC9367226

[86] Shen C, Jiang X, Li M, Luo Y. Hepatitis virus and hepatocellular carcinoma: recent advances. Cancers (Basel). 2023;15(2). 10.3390/cancers15020533. 10.3390/cancers15020533 PMC9856776 36672482

[87] Barsanti S, Salmi L-R, Bourgueil Y, Daponte A, Pinzal E, Ménival S. Strategies and governance to reduce health inequalities: evidences from a cross-European survey. Global Health Res Policy. 2017;2(1):18. 10.1186/s41256-017-0038-7. 10.1186/s41256-017-0038-7 PMC5683456 29202086

[88] Loud JT, Murphy J. Cancer screening and early detection in the 21(st) century. Semin Oncol Nurs. 2017;33(2):121–8. 10.1016/j.soncn.2017.02.002. 28343835 10.1016/j.soncn.2017.02.002 PMC5467686

[89] Lopez AM, Hudson L, Vanderford NL, Vanderpool R, Griggs J, Schonberg M. Epidemiology and implementation of Cancer prevention in disparate populations and settings. Am Soc Clin Oncol Educ Book. 2019;39:50–60. 10.1200/edbk_238965. 31099623 10.1200/EDBK_238965 PMC6556209

